# Live Vaccination Tactics: Possible Approaches for Controlling Visceral Leishmaniasis

**DOI:** 10.3389/fimmu.2014.00134

**Published:** 2014-03-31

**Authors:** Noushin Saljoughian, Tahareh Taheri, Sima Rafati

**Affiliations:** ^1^Molecular Immunology and Vaccine Research Laboratory, Pasteur Institute of Iran, Tehran, Iran

**Keywords:** *Leishmania*, visceral leishmaniasis, live vaccine, live attenuated vaccines, live non-attenuated vaccines

## Abstract

Vaccination with durable immunity is the main goal and fundamental to control leishmaniasis. To stimulate the immune response, small numbers of parasites are necessary to be presented in the mammalian host. Similar to natural course of infection, strategy using live vaccine is more attractive when compared to other approaches. Live vaccines present the whole spectrum of antigens to the host immune system in the absence of any adjuvant. Leishmanization was the first effort for live vaccination and currently used in a few countries against cutaneous leishmaniasis, in spite of their obstacle and safety. Then, live attenuated vaccines developed with similar promotion of creating long-term immunity in the host with lower side effect. Different examples of attenuated strains are generated through long-term *in vitro* culturing, culturing under drug pressure, temperature sensitivity, and chemical mutagenesis, but none is safe enough and their revision to virulent form is possible. Attenuation through genetic manipulation and disruption of virulence factors or essential enzymes for intracellular survival are among other approaches that are intensively under study. Other designs to develop live vaccines for visceral form of leishmaniasis are utilization of live avirulent microorganisms such as *Lactococcus lactis*, *Salmonella enterica*, and *Leishmania tarentolae* called as vectored vaccine. Apparently, these vaccines are intrinsically safer and can harbor the candidate antigens in their genome through different genetic manipulation and create more potential to control *Leishmania* parasite as an intracellular pathogen.

## Introduction

Several species of the protozoan genus *Leishmania* (*L*) causes a group of parasitic diseases called Leishmaniasis which generates different clinical symptoms from cutaneous (CL) to visceral leishmaniasis (VL). People living in Latin America, the Middle East, parts of Africa, Asia, and India have been affected by VL (also named Kala azar) which is a very deadly disease caused mainly by *L. (d) infantum*, *L. (d) donovani*, and *L. (d) chagasi* species. Kala azar causes a clinical syndrome identified by repetitive fever, anemia, hepatosplenomegaly, and a wasting disease accompanied with muscular atrophy and finally leads to death after all the sufferings. Sand flies that have already bitten infected dogs or humans transfer parasites to other humans through their bites. These *Leishmania* parasites have numerous survival strategies among which the intracellular replication is the most famous one and prevents the parasites from direct contact to the immune system by the surrounding host cells.

A Th1 type cytokine milieu causes the parasite load to clear while a Th2 type leads to the host’s susceptibility. Th1 cytokines can trigger macrophages, which are the major cells to destroy *Leishmania* parasites. To clear intracellular parasites, Th2 cells do not suffice since they induce a humoral response which has little or no effect on the parasites. Nowadays, controlling the disease depends mainly on chemotherapy as prophylactic or therapeutic vaccines are unavailable. VL chemotherapies have certain disadvantages such as the lengthy treatment time, costly drugs, and teratogenic effects. The reason for concern about resistance emergence is the long half-life of the chemotherapeutics (1–3).

**Figure 1:**
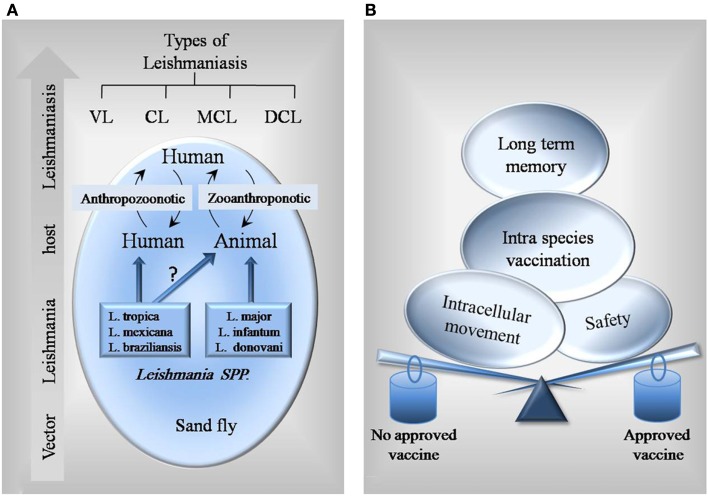
**Schematic figure of *Leishmania* interplay and different factors to consider in vaccine design. (A)** Most of *Leishmania* parasites such as *L. infantum* and *L. donovani* are known to have both human and animal hosts, so preventive vaccines could be designed for both. However, there is no identified reservoir animal host for some species like *L. tropica*. For these species, specific vaccines for human are needed. **(B)** Balance between different factors leads a vaccine to get approved.

The complex life cycle of *Leishmania* parasites, which consists of stages in animal or human and the sand fly vector, makes vaccine development more challenging (Figure 1A). An ideal antileishmanial vaccine should be able to solve current problems and limitations of other existing vaccines. As shown in Figure 1B, it should be safe, stable, reproducible, less risky, easily administered, stored and delivered, not reversible to infectious state, and able to induce long-term immunological memory and humoral and cellular responses.

In CL form of disease, the life-long protection is generated against the same disease and this is the fact that promises the feasibility of a vaccine. Deliberate infection with parasites at hidden body sites where scars ensue is a method that has been exploited in the leishmanization (LZ) practices of the last century (4). Nations, particularly in the Middle East, have successfully used the strategy for mass prevention of CL, but it need to improve due to persistence of monthly adverse effects and local lesions in 2–3% of cases (5).

In the late 1930s, researchers in Brazil showed that killed parasites were efficient when used as therapeutic as well as prophylactic; afterward first generation vaccines were produced from the whole killed *Leishmania* parasites (6). For many years, these vaccines were tested either alone or combined with different adjuvants. So far, killed parasites had no enough efficacy as a potent vaccine to prevent disease, although they have demonstrated well-tolerated safety profiles (7).

First generation vaccines produced from VL *Leishmania* species have had no chance to be tested in clinical trials, since most vaccine studies have concentrated on CL. What have been included for the progression of *Leishmania* second generation vaccines are recombinant proteins, poly-proteins, DNA vaccines, and combinations thereof. In experimental infection systems, not only defined single molecules, but also multi-component vaccines have shown protection against VL. Coler et al. worked on LEISH-F1 + MPL-SE, which consisted of three recombinant *Leishmania* poly-protein (TSA–LmSTI1–LeIF), in association with monophosphoryl lipid and squalene as adjuvants (MPL-SE) (8). The synthetic RAPSODI^1^ and two other DNA vaccines are in preclinical trials in Europe; one is being developed based on a viral vector by Paul Kaye (York University, UK) and another, LEISHDNAVAX^2^, by Mologen (Berlin, Germany) using a new technology named minimalistic immunogenically defined gene expression (MIDGE) to deliver selected *Leishmania* antigens; the latter can be used either solely or accompaniment to a synthetic adjuvant – double stem loop immunomodulator (dSLIM).

**Figure 2:**
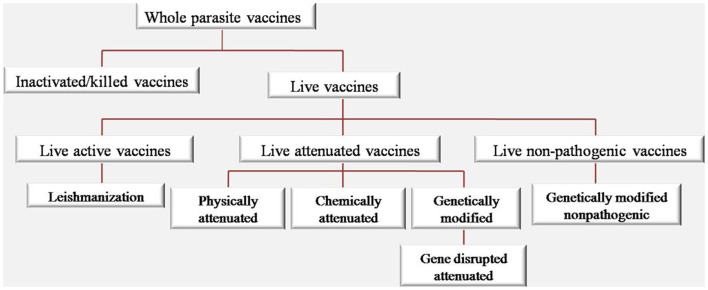
**Categorization of vaccine types based on whole *Leishmania* parasite**.

It is believed that if a candidate vaccine could stimulate immune system more similar to the natural disease, we will have a more efficient immune response. As the success of smallpox, measles, mumps, and rubella vaccines indicate that live attenuated vaccines are the touchstone for protection against their specific causing pathogen. As shown in Figure 2, different approaches were used based on whole parasite vaccine ranging from live active *Leishmania* vaccine (LZ) to live non-pathogenic vaccines.

Some attenuated strains were also developed through different approaches such as physical, chemical, and genetically attenuation. Much interest has been arisen in the development of genetically attenuated parasite vaccines due to the knowledge obtained in potential parasite virulence factors and the increased understanding of the antigens participating in immunity acquisition. Targeting and deleting genes that encode virulence factor genes essential for intracellular survival is the major general approach toward genetic attenuation of *Leishmania* parasites. Recently, there are few successful reports about live attenuated *Plasmodium* through genetical modification that can elicit long-lasting memory protection by producing antibodies and cellular immune responses (9). Interestingly, in recent human clinical trial using *Plasmodium falciparum* genetically attenuated parasites (*Pf*GAP) as vaccine on volunteers showed the first in human proof of concept of this strategy that could inhibit the expansion of disease by decreasing the sporozoites (10).

Using BCG as a vaccine against *Mycobacterium tuberculosis* infection is a method which is comparable with utilizing non-pathogenic *Leishmania* species, such as a lizard parasite *L. tarentolae*, to develop live non-pathogenic parasites as VL vaccines. Although *L. tarentolae* can infect mammalian cells and change to amastigotes, it does not cause any disease or clinical symptoms in either mouse or hamster models (11, 12). Furthermore, due to general feasibility of human vaccination with live *Salmonella* and *Lactococcus* expressing exogenous antigens, they could serve another means to develop vaccine against leishmaniasis.

In this review, we have limited our scope to all types of live vaccinations against leishmaniasis and have considered them as vaccine candidates against leishmaniasis.

## Leishmanization (Live Active Vaccines)

In the past, mothers used to expose their children’s arms to be bitten by sand flies because they knew by experience that this would protect them from the severe disease in future. LZ was accepted in Israel and Russia after a method for axenic culture of the parasites was established (13). Using LZ was stopped because of HIV spreading, the use of immunosuppressive drugs, ethical reasons, uncontrolled permanent skin lesions, parasite persistence, and the inoculum quality control problems. The only usage of LZ at the present time is found in one of the endemic country, Uzbekistan, which is licensed and in Iran its efficacy is in human trials. Scientists are trying to improve the safety of this practice because it is the only way against *Leishmania* that has proved efficient in humans. The severity of primary lesions is reduced and wound healing is accelerated by including killed parasites in the inoculums and using adjuvants that improve quick immune responses (14, 15).

## Live Attenuated Vaccines

Different methods such as physical attenuation: long-term vitro cultures (16), temperature sensitivity (17), γ-attenuation (18), and chemical attenuation: chemical mutagenesis (19), and parasite culture under drug pressure (20) were used to develop attenuated strains.

Instead, using a targeted gene disruption strategy can lead to a genetic alteration of the *Leishmania* genome that could help identifying essential genes for survival and/or virulence (21–27) (Table 1). Generally speaking, live attenuated organisms are quite acceptable for vaccination because, first, such vaccines render native antigen into cells and improve activation of antigen-presenting cells at the same time by imitating the natural course of infection, which will lead to an optimal polarization of CD4^+^T cells (28); second, the memory repertoire of the immune system is increased since a collection of complete antigens is delivered (in comparison with subunit-defined vaccines); and third, they assure antigen persistency by generating prolonged sub-clinical infection. Then, generation of antigen-specific effector and memory cells which react soon after infection may be allowed (29). Substantial protection in murine models against challenge has been conferred by attenuated strains, but potential for reversion is possible for ever, which makes them inappropriate for use in human vaccination. Actually, risk of subsequent reactivation, especially in HIV/*Leishmania* co-infection, is raised by the persistence of asymptomatic *Leishmania* infections. In addition, a loss of effectiveness for protective immunity can be resulted from physical and chemical attenuation, either because a sub-clinical infection cannot be formed by such strains or because they do not express critical antigen epitopes anymore (30). Although the experimental results have been promising so far, there are still some safety points that need to be considered in relation to the use of genetically attenuated parasites as vaccines. Prolonged immunity after re-infection induces live attenuated vaccines through maintaining a low level asymptomatic infection. Since the persistence of antigen is essential to generate effective memory responses to *Leishmania*, the establishment of sub-clinical infection is considered quite valuable. Patients who are immunocompromised (e.g., after HIV infection) have shown reactivation of *Leishmania*. This is the reason why it is necessary that the safety of attenuated parasites that cause a sub-clinical infection should be carefully investigated.

**Table 1:** **Live attenuated vaccines against leishmaniasis**.

Attenuated vaccine form	Species	Animal model	Result	Reference
**PHYSICALLY ATTENUATED**				
Long-term cultured	*L. major*	C57BL/6 and BALB/c	C57BL/: completely resistant; BALB/c: partially protection, persistent low-grade cutaneous disease	(16)
	*L. tropica*	
	*L. major*	BALB/c	Protection	(31)
	*L. chagasi*	BALB/c	No protection	(30)
	*L. amazonensis*	C57BL/6	Smaller lesions, ↑ IFN-γ, ↓parasite load	(32)
Temperature sensitivity	*L. braziliensis*	BALB/c	Protection	(17)
Radio-attenuated	*L. major*	CBA	Resistance to subsequent infection with *L. mexicana*	(33)
Gamma irradiation	*L. major*	CBA and BALB/c	Protection against homologs and heterologous challenge	(18)
**CHEMICALLY ATTENUATED**				
With *N*-methyl-*N*′-nitro-*N*-nitrosoguanidine	Avirulent *lpg*^−^deficient *L. major*	BALB/c	↓Lesion size, resistance to a subsequent challenge	(19)
Culturing *in vitro* under gentamicin pressure	*L. mexicana* and *L. major*	BALB/c	No lesions, Th1-like responses	(20, 34, 35)
			↓Th2 responses, modulate the host immune response	
			Significant protection	
Culturing *in vitro* under gentamicin pressure	*L. infantum*	Dogs	No clinicopathological abnormalities	(36–38)
			↑ IFN-γ, ↓IL-10, ↑ IgG2	
			↑ CD4+ and CD8+ T cells	
**GENETICALLY ATTENUATED**				
*dhfr-ts* Null mutant	*L. major*	BALB/c	Protective	(21)
Cysteine proteinase-deficient mutant	*L. mexicana*	BALB/c, C57BL/6, CBA/Ca	Immune response modulation, Th1 response	(24)
*dhfr-ts* Null mutant	*L. major*	Monkeys	No protection	(39)
lpg2-	*L. major*	BALB/c	Protection, no strong Th1 response	(26)
Cysteine proteinase-deficient mutants	*L. mexicana*	Hamsters	Delayed disease onset	(40)
			↓Smaller lesions	
			↓Parasite burden, ↓IL-10 and TGF-beta, and protection	
LiSIR2(±) mutant	*L. infantum*	BALB/c	↑ IFN-γ/IL-10 ratio, ↑ NO, protection	(27)
Phosphomannomutase-deficient mutant	*L. major*	BALB/c	↓IL-10 and IL-13, ↑ CD44hi T cell recruitment	(41)
			Protection	
LdCen1(^−/−^) mutant	*L. donovani*	BALB/c SCID hamsters	↑ IFN-γ, IL-2, TNF, ↑ IgG2a, ↑ IFN-γ/IL-10 ratio, ↑ NO, Th1 response, long-lasting protection in hamsters	(42)
HSP70-II null mutant	*L. infantum*	BALB/c	↑ NO, type 1 responses	(43)
Ldp27(^−/−^) mutant	*L. donovani*	BALB/c	Long-term protection	(44)
cLdCen(^−/−^) mutant	*L. donovani*	Dogs	↑ Type 1, ↓Type 2	(45)
			↑ Immunogenicity	

## Live Physically Attenuated Vaccines

It was shown by Mitchell et al. that long-term cultured promastigotes of *L. major* and *L. tropica* isolates could not cause lesions after cutaneous injection to mice (16). One year later, the effect of long-term cultivation of *L. donovani* promastigotes on cultured mouse and hamster macrophages *in vitro* was evaluated by Nolan et al. In a period of 48 days, the number of amastigotes derived from long-term promastigote cultures decreased only slightly in mice but rapidly in hamsters (46). In another experiment, 8 weeks after infection, long-term cultured *L. amazonensis* promastigotes induced smaller lesions, produced higher IFN-γ, and made smaller parasite load compared to the short-term cultured counterparts. Macrophages infected by long-term cultured parasites expressed high level of chemokine CXCL10 mRNA, which might activate these cells to kill the parasites (32). Nevertheless, there are several similar trials which led to ineffectiveness, such as long-term *in vitro* culture of *L. chagasi* that did not create protective immunity (30). Using temperature-sensitive avirulent parasite clones, the immunized susceptible BALB/c mice were successfully protected against *L. braziliensis* (17). Radio-attenuation, first introduced in 1974 by Lemma et al., is another physical approach for preparation of *Leishmania* vaccine (47). The resistance of CBA mice to subsequent infection with *L. mexicana* is highly increased by administration of radio-attenuated *L. major* vaccines (33). In another experiment, gamma irradiation of *L. major* elicited a high degree of protection against homologs and heterologous challenge in CBA and BALB/c mice (18). Although most of these methods showed promising protective effects, they were not further used in research studies of vaccination against *Leishmania* species, due to safety issues regarding incomplete inactivation and reversion of infectivity (Table 2).

**Table 2:** **Properties of different types of live vaccines based on whole organisms**.

Type of live vaccines	Benefits	Concerns
Leishmanization	Life-long protection	No safety (48) and high risk (49)
Live non-attenuated vaccines	Almost successful and immunity (48, 50)	Exacerbate the disease, reversion to virulence, large persistent lesions, psoriasis, and immunosuppression
		Not reproducible (48), no efficacy, and no standardization and quality control (48, 50)
		Risk of HIV transmission
Physically attenuated	Cheaper	No safety, high risk, incomplete attenuation, no efficacy, not reproducible, non-specific attenuation (51), and reversion to virulence (51)
		Not acceptable for humans (50), risk of DNA damage
Chemically attenuated	Cheaper	No safety, high risk, incomplete attenuation, no efficacy, not reproducible, risk of random mutations, non-specific attenuation (51), and reversion to virulence (51)
Genetically attenuated	Safer, more stable (48)	Reversion to virulence
	Natural course of infection (50)	Presence of antibiotic resistance genes (52); storage and delivery
Non-pathogenic organism	Safer (52), cross-reactivity between species (48), induce both humoral and cellular response (48)	Not appealing prospect (48) Possible reversion to virulence or reactivation (52) Presence of antibiotic resistance genes (52) Storage and delivery
	Lower risk of reversion to the virulent phenotype, highly immunogenic	
	Natural course of infection	
	For some easy administration	

## Live Chemically Attenuated Vaccines

To immunize susceptible BALB/c mice against challenge with virulent *L. major*, Kimsey et al. used an avirulent clone of *L. major* which was prepared after several *in vitro* treatments of a virulent population of *L. major* with the mutagen, *N*-methyl-*N*′-nitro-*N*-nitrosoguanidine (MNNG), and could control lesion size in the challenge mice model (19). It has been shown that an avirulent lipophosphoglycan-deficient *L. major* clone is able to elicit resistance to a subsequent challenge with virulent *L. major* while it is unable to produce cutaneous lesions in susceptible BALB/c mice (19). Similarly, in another experiment, avirulent lipophosphoglycan-deficient *L. donovani* parasites could not generate visceral infection in hamster model after inoculation through the intra cardiac route, contrary to virulent *L. donovani* (53). Different species of *Leishmania* have been attenuated by culturing *in vitro* under gentamicin pressure successfully such as *L. mexicana*, *L. major*, *L. infantum*, and *L. donovani*. While wild-type (WT) parasites survived and multiplied, the attenuated strains were able to invade but they neither could survive within bone marrow-derived macrophages *in vitro* nor induce cutaneous lesions in BALB/c mice after about 12 weeks. High level of protection was induced in mice against challenge with WT parasites by both attenuated lines of *L. mexicana* and *L. major* (20). This was accompanied by a CD4^+^Th1-like response in BALB/c mice that was shown by the cytokine profile of their WT *L. mexicana* promastigotes-stimulated splenocytes (34). Growth of the WT parasites was excessively controlled in experiments wherein mice were simultaneously inoculated (either at the same site or on separate sites) with attenuated and WT parasites, showing that the attenuated parasites have a possible therapeutic role. Comparing dogs infected with either WT *L. infantum* or gentamicin-attenuated *L. infantum* H-line, no pathological abnormalities were observed in the latter group, which induced significantly higher IFN-γ and lower IL-10 levels with the highest levels of IgG2 subclass in their sera (37). Also, proliferation of mononuclear cells is associated with cellular immunity in immunized dogs (38). However, in addition to the difficulty of large-scale production of these physically attenuated vaccines and their delivery to the field in appropriate conditions, the major drawback is their loss of effectiveness for protective immunity due to their inability to form sub-clinical infection and express critical antigen epitopes (30) (Table 2).

## Live Genetically Attenuated Vaccines

Development of transfection technology has acted as a powerful reverse molecular genetics tool for genetic modifications in the last two decades. Gene delivery into such unicellular pathogens as *Leishmania* has created a great revolution in making genetically defined vaccines through knocking out/in certain genes. DNA delivery by physical methods is a very efficient and easy system; DNA fragments are best transferred into parasites nuclei by transfection through electroporation (54). A linearized construct containing antibiotic resistant genes should be integrated into the genome through homologous recombination (HR) to remove a gene. This allows a DNA sequence transfer into the locus of interest in the *Leishmania* genome using two flanking sequences in both sides of the gene (54).

To generate an absolute knockout, the *Leishmania* parasite needs a second construct to bear another antibiotic resistant gene to replace the second gene alleles. The cell phenotype is altered by this manipulation and new parasite features are naturally transferred to the next generations through inheritance. Controlling the gene in its new genome location is the most crucial concern in gene targeting because it may affect the normal gene functionality in both sides of the target. Therefore, gene entrance location is very important and should be confirmed by molecular genetics methods although *Leishmania* genome is relatively easy to manipulate. Phenotypic changes (e.g., morphology, growth, infectivity) of the manipulated parasite after each transfection are other critical issues that need to be studied.

In this direction, one of the first experiments to vaccinate mice against challenge with virulent *L. major* was done by Titus et al. (21) using *dhfr-ts* null mutant of *L. major* obtained by gene targeting. Although it could not produce protective immunity in primates and needed further improvement for vaccine application (39), it could elicit considerable resistance phenotype after BALB/c mice challenge with virulent *L. major* (21). *L. mexicana* mutants lacking cysteine proteinase genes generated by targeted gene disruption were tested on murine and hamster models in another attempt and could induce delayed disease onset, smaller lesions, and lower parasite burden in mice and hamsters (24, 40). Thus, the idea of the feasibility of using genetically attenuated live *Leishmania* to achieve protective immunity was supported by such findings. Uzonna et al. showed that highly susceptible mice could be protected against virulent challenge without inducing a strong Th1 response when vaccinated with phosphoglycan-deficient *L. major* (26). A much less capacity compared to the WT parasites was shown in *L. donovani* BT1 null mutant for inducing infection in mice, and those susceptible to infection against *L. donovani* challenge attained protective immunity (25). Silvestre et al. showed that SIR2-deficient (silent information regulatory 2) *L. infantum* induced a clear IFN-γ/IL-10 pattern that is associated with protection patterns (27). In another study, susceptible BALB/c mice showed protection against infection when vaccinated with avirulent *L. major* phosphomannomutase-deficient parasites (41). Kedzierski et al. concluded that the factors that play essential parts in eliciting protection against *Leishmania* are increase in the number of T cells, their rapid recruitment to lymph nodes upon infection, and lower production of IL-13 and IL-10 (which leads to high IFN-γ/IL-10 ratio). It was shown in 2009 that live attenuated *L. donovani* parasites by gene disruption of centrin gene (LdCen1^−/−^) could be live, safe, and induce protection in susceptible BALB/c mice, immunocompromised severe combined immunodeficiency (SCID) mice and hamsters. Infection with *L. braziliensis*, which causes mucocutaneous leishmaniasis, could be prevented if mice were immunized with LdCen1^−/−^ (42). It was shown by Fiuza et al. that strong antibody production, Type 1 polarization, and Type 2 inhibition could be induced by LdCen^−/−^ vaccine in dogs, as an important reservoir host (45). Dey et al. have shown that *L. donovani* mutant of amastigote-specific protein p27 knockout (Ldp27^−/−^) as live attenuated parasites are safe, induce protective immunity, and can provide protection against homologous and heterologous *Leishmania* species (44). Carrion et al. believe that the ability of a safe genetically modified *L. infantum* mutant, which lacks both HSP70-II alleles (ΔHSP70-II), provide protection against *L. major* infection in BALB/c and can lead to the production of high levels of NO, type 1 immune responses, and IgG subclass analyses in mice (43). However, there are some limitations for their extensive use such as safety constraints due to reversion to virulent form especially in immunosuppressed individuals and manufacturing concerns.

## Live Non-Pathogenic Vaccines

Utilization of non-pathogenic species as *Salmonella enteric*, *Lactococcus lactis*, and *L. tarentolae* to develop live attenuated parasites as VL vaccines is another approach. This approach has shown enhanced antigen presentation and potent Th1 response similar to BCG, a successful vaccine against *M. tuberculosis* infection (Table 3). These methods can be further refined through the use of their recombinants expressing antigens of virulent *Leishmania* spp. In general, the most promising strategic alternative against VL can be claimed to be the use of live, non-pathogenic/genetically engineered strains of these species.

**Table 3:** **Live non-pathogenic vaccines against leishmaniasis**.

Vaccine form	Species	Animal model	Result	Reference
***SALMONELLA ENTERICA***				
*S. typhimurium* aroA-+gp63 (SL3261-gp63)	*L. major*	CBA	↑ T helper 1 protection	(55)
*S. typhimurium* aroA- aroD-+ +gp63 (GID101)	*L. major*	BALB/c	↑ Th1 subset of CD4+ T cells protection	(56)
*S. typhimurium* aroA- aroD- (BRD509), +MIF, IL-2, IFN-γ, or TNF-alpha (GIDMIF, GIDIL2, GIDIFN, and GIDTNF)	*L. major*	BALB/c	Limited lesion development	(57)
			↑ Nitric oxide synthase (iNOS)	
			↓ Parasite loads, protection	
*S. typhi* delta aroC, delta aroD (CVD 908), ++gp63 (SL3261-gp63)	*L. m. mexicana*	F1 (BALB/cXC57BL/6)	T cell-mediated response	(58)
			Protection or resolution of the infection	
DNA-*Salmonella*+ +LACK antigens primer-booster	*L. major*	BALB/c	↑ Th1, ↑ IFN-γ, ↑ IgG2a	(59)
			Protection	
*S. typhimurium* SL3261+ +LinJ08.1190 and LinJ23.0410	*L. major* and *L. donovani*	BALB/c	↑ Resistance against visceral leishmaniasis	(60)
***LACTOCOCCUS LACTIS***				
A2-expressing *Lactococcus lactis*	*L. donovani*	BALB/c	↑ Liver parasitemia	(61)
			↑ Antibody titers, critical influence on the immune response	
*Lactococcus lactis* co-expressing LACK and IL-12	*L. major*	BALB/c	↓ Parasite burden	(62)
			↑ Th1 response	(63)
			Partially protection	
			Delay in footpad swelling	
***LEISHMANIA TARENTOLAE***				
*L. tarentolae*	*L. donovani*	BALB/c	↑ *Leishmania*-specific TH1 immune response	(12)
			Protection	
Recombinant *L. tarentolae* expressing A2 gene	*L. infantum*	BALB/c	Intraperitoneal administration: ↑ IFN-γ, ↓ IL-5, ↑ Th1, protection	(64)
Recombinant *L. tarentolae* expressing A2–CPA–CPB^−CTE^	*L. infantum*	BALB/c	↑ IFN-γ, ↓ IL-10, ↑ NO	(65)
			↑ IFN-γ/IL-10 ratio	
			↓ Parasite burden, protection	

### Salmonella enterica

*Salmonella* (*S*) are intracellular pathogens that upon entrance to human macrophages induce a viscerotropic immune response similar to *Leishmania*. Development of live *Salmonella* vaccines as a method for delivering heterologous antigens was discussed for the first time in 1987 (66). The important advantage of using attenuated *Salmonella* for vaccination against VL is their low production cost, storage at room temperature, and their oral, needle-free application if rehydrated. Since orally administered live attenuated *Salmonella* spp. that express heterologous antigens are safe and highly immunogenic, they are promising candidates; they can elicit prolonged, protective, systemic, and mucosal immune responses against the heterologous pathogen (67).

*In vivo* inducible promoters and optimized expression systems are used to construct novel attenuated *Salmonella* vaccines that deliver antigens and show a host protective effect in small rodent models of VL. Live *Salmonella* needs more studies to promote their further application.

Furthermore, for delivery and expression of vaccine antigens in the host, several attenuated lines of *S. typhimurium* have been generated. For more safety, more than one attenuating mutation can be incorporated in a vaccine. Several derived antigens (target carbohydrate, protein) or epitopes from different pathogens, viruses, bacteria, and eukaryotic parasites are expressed by combined *Salmonella* vaccines in the form of capsules, fimbria, or flagellum, either within or on the surface of the cell (68). A very significant resistance was developed against a *L. major* challenge infection by the mice that had been orally immunized with gp63-transformed *S. typhimurium* (55, 56). *S. typhimurium* derivatives (GIDMIF, GIDIL2, GIDIFN, and GIDTNF) expressed cytokines *in vitro* under anaerobic conditions. They were stably colonized in orally immunized BALB/c mice more than 14 days and showed protective effect which correlated with the induction of inducible nitric oxide synthase (57).

Lange et al. showed that production of IFN-γ could induce protection against *L. major* infection in susceptible BALB/c mice and were enhanced as a result of using LACK antigens in DNA-*Salmonella* primer-booster vaccination compared to that with the DNA alone (59). In a recent study, Schroeder et al. identified two novel candidate vaccine antigens (LinJ08.1190 and LinJ23.0410) by reverse vaccinology and utilized them in the construction of live *Salmonella* carriers against VL, which reduced visceralization considerably and increased resistancy against *L. donovani* infection in susceptible BALB/c mice (60).

### Lactococcus lactis

*Lactococcus lactis* is a Gram-positive, non-pathogenic, non-colonizing lactic acid bacterium (69), which is industrially important and is frequently used in the preparation of fermented foods and dairies; FDA has given it a generally recognized as safe (GRAS) status [(70); aminopeptidase enzyme preparation derived from *L. lactis* (21CFR184.1985)].

It has been used as a live bacterial delivery vector for more than 10 years (71) and scientists are being encouraged to use it as a live vaccine against leishmaniasis. A2-expressing *L. lactis* live vaccines have been generated and evaluated by Yam et al. against *L. donovani* in BALB/c mice. This A2 anchored to the cell wall has a critical influence on the immune response; this sub-cellular location of antigen expression causes the highest reduction in liver parasitemia, induces the highest level of antigen-specific antibody titers which is seen at both low- and high-dose *L. donovani* parasite challenges (61). In another study of this group it was shown, using LACK- and IL-12-expressing *L. lactis*, that subcutaneous immunization against *L. major* infection delays footpad swelling, indicating the necessity for co-administration of *L. lactis*/sec IL-12 (secreting IL-12) as a Th1-inducing adjuvant (63). Again in another study, the same group showed that if live *L. lactis* secreting both LACK and IL-12 was used, oral immunization was the only regimen that could protect BALB/c mice partially against *L. major* infection (62). The *L. lactis* line generated in these studies provides an attractive cornerstone for further research on live-based vaccines against leishmaniasis and other pathogens.

### Leishmania tarentolae

Recently, the use of a non-pathogenic *Leishmania* vector (*L. tarentolae*) was suggested by Breton et al. (12) as a vaccine candidate against leishmaniasis which is known as non-pathogenic for human since it is not able to generate any manifestation of human leishmaniasis. Although this parasite is non-pathogenic in either mouse or hamster models because it lacks any clinical symptoms, it can infect mammalian cells and transform into amastigotes (72). Genome sequence analyses have revealed that this parasite is syntonic to the three sequenced pathogenic *Leishmania* species (*L. major*, *L. braziliensis*, and *L. infantum*) and that more than 90% of the approximately 8200 genes are shared by all the species. Nevertheless, some of the essential genes that are relevant to pathogenicity in pathogenic strains or expressed in amastigote form are absent in *L. tarentolae* or were in variable copy number. This supports the idea that some of these genes are possible to be associated with reduction of pathogenic capacity in *L. tarentolae* and make it an intracellular parasite and its diminished pathogenic potential to humans. As an example, the amastin family, especially the delta group as just two copy number in *L. tarentolae* while high copy numbers (12–25) are found in the pathogenic species (73). Why *L. tarentolae* cannot replicate efficiently in mammalian macrophages can be explained by the absence of these proteins. It has been shown in experimental vaccine trials that a single intra peritoneal immunization of *L. tarentolae* elicited a protective immune response against *L. donovani* in susceptible BALB/c mice; it was concluded that it was a result of an enhanced antigen presentation and potent Th1 immune response (12). Since *L. tarentolae* is a safe vector for use as a vaccine, it can be more effective anti-*Leishmania* vaccine by genetic manipulation in order to induce transgenic *L. tarentolae* which expresses certain immunodominant *Leishmania* antigens.

Effort has also been made to use *L. tarentolae* as a specific deliver and expression system for *Leishmania* antigens in host. The *L. donovani* A2 antigen was expressed in *L. tarentolae*, which normally lacks this protein (74) and used as a vaccine strain in an experimental mouse model. The susceptible mice were protected against *L. infantum* infection through vaccination following high levels of IFN-γ were produced (64). In addition, *L. tarentolae* can be used as a promising live vaccine vector against intracellular pathogens. This idea was examined for the first time in an experiment using a recombinant *L. tarentolae* expressing HIV-1 Gag protein as a candidate HIV-1 vaccine. It was shown that the vaccine induces a strong cell-mediated immunity in BALB/c mice and decreases HIV-1 replication in an *ex vivo* condition (75). Also, a novel live vaccine using recombinant *L. tarentolae* expressing E7 protein for the protection of mice against HPV-associated tumors was produced and evaluated (76). It is worth mentioning that this vaccine showed the best protection and minimum tumor size among all other groups against TC-1-induced tumors (76).

Our team produced a recombinant *L. tarentolae* expressing the A2–CPA–CPB^−CTE^ tri-gene fusion that are three important vaccine candidate antigens of *L. infantum*, as a new live vaccination strategy against visceral form of leishmaniasis in two-modalities, namely DNA/live and live/live vaccination in BALB/c mice. We demonstrated how prime-boost (DNA/live) strategies using recombinant *L. tarentolae*-based vaccines elicited promising immunization against a high-dose virulent *L. infantum* challenge (65). We also tested live/live *L. tarentolae*-A2–CPA–CPB^−CTE^ prime-boost vaccination regime in hamsters and showed that it represented an appropriate animal model in the discovery of potential antigens that could be used in the control of canine VL (unpublished data). The parasite loads in both visceral organs were controlled in the vaccinated hamsters reaching a negligible level by day 56 post challenge, demonstrating its strong vaccine potential. Five weeks after infection by *L. infantum*, hamsters that had received the live vaccine produced higher levels of anti-*L. infantum* lysate antibodies than those injected with PBS control.

In another attempt, we tested the efficacy of a novel combination of established protective parasite antigens expressed by *L. tarentolae* together with saliva antigens as a vaccine strategy against *L. major* infection. Different DNA/live and live/live prime-boost vaccination modalities with live recombinant *L. tarentolae* stably expressing cysteine proteinases (type I and II, CPA/CPB) and PpSP15, an immunogenic salivary protein from *Phlebotomus papatasi*, a natural vector of *L. major*, were tested in both susceptible BALB/c and resistant C57BL/6 mice. In both strains of mice, the strongest protective effect was observed when priming with PpSP15DNA and boosting with PpSP15 DNA and live recombinant *L. tarentolae* stably expressing cysteine proteinase genes (accepted in PLoS NTD, 2014).

Regarding vaccine development in dogs, with lack of enough knowledge about canine leishmaniasis and canine immunity, it is almost impossible to predict the results obtained from the mouse and hamster models, if vaccine candidates can work in dogs. Therefore, it is essential to do more studies on dogs for both new vaccine candidates and immune response analyses. Whether or not protection will be achieved, results of such tests would be valuable for the advancement of knowledge about canine leishmaniasis and giving a guided direction to future protection strategies. It is worth to mention that our group is testing the genetically knock in *L. tarentolae* expressing the A2–CPA–CPB^−CTE^ tri-gene fusion as a live vaccination strategy with different modalities in outbreed dogs.

## Conclusion

Unlike most other pathogens, *Leishmania* never clears fully by immune system and we do not need sterile immunity. The important issue for maintenance of immunity is believed to be the presence of small number of live parasite in the host. Live replicating parasites or just persistent antigens are believed to be important for the maintenance of effector memory like T cells but not for central memory T cells. It has been reported that the quality of memory cells in the presence and absence of live parasite are different in CL (77). In the case of VL, persistence of parasite antigen is important for generating antigen-specific effector T cells, although more depth studies are required to be analyzed in the case of non-pathogenic and/or genetically attenuated *Leishmania* parasite (44). During *Leishmania* infection, we need a methodical understanding of how the immunological memory is generated and maintained, what the sustained long-term protective immune responses are, and through what mechanisms vaccines stimulate protective immunity. An ideal anti-*Leishmania* vaccine must maintain constant turnover of *Leishmania*-specific memory cells in vaccinated host, otherwise repeated booster injections would be required (78).

Immune response to *Leishmania* is very complicated and for wisely designing vaccines we need to know which T cell determinants act as IFN-γ inducer (CD8+ or CD4+ T cell) and are essential for long-term immunity. Long-lasting protective immunity induced by vaccination is a pragmatic goal for control of parasitic infections. In LZ, the only successful strategy that has been used to induce resistance to cutaneous leishmaniasis, after obviation of the infection, individuals are resistant to re-infection. It is now clear that in mice infected with WT parasites, heterogeneous memory CD4+ T cell pool contain two subsets, specified by their expression of the LN-homing molecule CD62L, one of them, effector memory T cells, has the characteristics of effector cells (CD62Llo) and the other one, central memory T cells, act as a repository of antigen-specific T cells (CD62Lhi) and can extend upon rechallenge, differentiate into effector T cells, and refill the effector cell population (79, 80). The latter which expressed CD62L and lodged to the lymph nodes, expand early after infection with *L. major* (81). However, the first population of cells CD62Llo effector T cells could intercede resistance faster than the CD62Lhi central memory T cells (80). In other words, at providing immunity to rechallenge in leishmaniasis central memory CD4+ T cells that could be maintained without persistent parasites were less effective. This observation indicates that for immunity maintenance and providing long-term immunologic memory, persistent parasites may well be needed (82). Therefore, on this basis the idea of using live vaccine either in attenuated or non-pathogenic form is strengthened.

It is preferred that attenuating process of *Leishmania* strains for the production of live vaccine be done selectively (i.e., only in intracellular form or amastigotes); this will allow the cultivation of promastigotes in large-scale. Attenuation needs to be optimized so that the power of live parasite vaccines can be improved, but it should be noted that reversion of these parasites to the virulent form restricts their use. In other words, returning back to virulence is also probable; hence, the need for the production of new safer live vaccine vectors such as non-pathogenic *L. tarentolae* harboring immunogenic antigens that can enhance antigen presentation and elicit potent immune responses, without any risk of disease development in humans, becomes obvious. Using *L. tarentolae* as non-pathogenic vector is promised because of its safety and easy adaptation to mammalian system. Also, it has not the ability to revert to pathogenic form due to its non-pathogenic intrinsic property (11, 12). But what is certain is that *L. tarentolae* could not long survive in the mammalian cell, so it is best to think of some strategies to prolong its life there. Finally, there are still several obstacles for utilization of live non-pathogenic *Leishmania*, such as lyophilization and storage of this organism, which need special attention and serious research.

## Conflict of Interest Statement

The authors declare that the research was conducted in the absence of any commercial or financial relationships that could be construed as a potential conflict of interest.

## Footnotes

^1^http://www.fp7-rapsodi.eu/

^2^http://www.leishdnavax.org/
